# Effect of initial body orientation on escape probability of prey fish escaping from predators

**DOI:** 10.1242/bio.023812

**Published:** 2018-06-26

**Authors:** Hibiki Kimura, Yuuki Kawabata

**Affiliations:** Graduate School of Fisheries and Environmental Sciences, Nagasaki University, Bunkyo-machi, Nagasaki 852-8521, Japan

**Keywords:** Attack angle, C-start, Escape response, Fast-start, Kinematics, Predator-prey interaction

## Abstract

The kinematic and behavioral components of the escape response can affect the outcomes of predator-prey interactions. For example, because sensory perception range can have spatial bias, and because turn duration before the initiation of escape locomotion can be smaller when prey is oriented away from predators, the prey's body orientation relative to a predator at the onset of the escape response (initial orientation) could affect whether prey successfully evade predators. We tested this hypothesis by recording the escape responses of juvenile red sea bream (*Pagrus major*) to the predatory scorpion fish (*Sebastiscus marmoratus*). Flight initiation distance tended to be small when prey were attacked from behind, suggesting that prey have spatial bias in detecting attacking predators. An increase in flight initiation distance increased escape probability. An increase in initial orientation decreased turn duration and increased escape probability when the effect of flight initiation distance was offset. These results suggest that initial orientation affects escape probability through two different pathways: changes in flight initiation distance and turn duration. These findings highlight the importance of incorporating initial orientation into other studies of the kinematics of predator-prey interactions.

## Abstract (precis)

**Summary:** Our predator-prey experiments reveal that prey's initial body orientation relative to a predator affects the flight initiation distance and turn duration of prey and consequently affects escape probability.

## INTRODUCTION

When exposed to sudden predation threats, most animals exhibit escape responses that include turning swiftly and accelerating forward (Bulbert et al., 2015; Camhi et al., 1978; Webb, 1986). Since the escape response is crucial to survival and hence to the fitness of the species, numerous studies have been conducted to elucidate the environmental and internal factors that affect the behavioral and kinematic components of the escape response (e.g. flight initiation distance, escape trajectory, turning speed, acceleration, etc.) (Bateman and Fleming, 2014; Cooper, 2006; Cooper et al., 2007; Domenici, 2010; Meager et al., 2006).

Previous theoretical studies have shown that the outcome of the escape response is dependent on flight initiation distance, predator and prey speeds, and the escape trajectory (Arnott et al., 1999; Broom and Ruxton, 2005; Domenici, 2002; Weihs and Webb, 1984). Interestingly, however, these studies have not incorporated the prey's initial body orientation with respect to the predator (hereafter, initial orientation) and the prey's turning speed, despite the fact that turning requires additional time prior to the initiation of escape locomotion (King and Comer, 1996) and that initial orientation affects the turn angle (Cooper and Sherbrooke, 2016; Eaton and Emberley, 1991; Kawabata et al., 2016). Empirical studies show that turning speed, as well as the above variables, affects predator evasion (Dangles et al., 2006; Fuiman, 1993; Scharf et al., 2003; Stewart et al., 2013; Walker et al., 2005; Webb, 1982; Webb and Zhang, 1994), however, as far as we aware, except for one study (Stewart et al., 2013), no research has been conducted on the effect of initial orientation on escape probability.

**Fig. 1.**
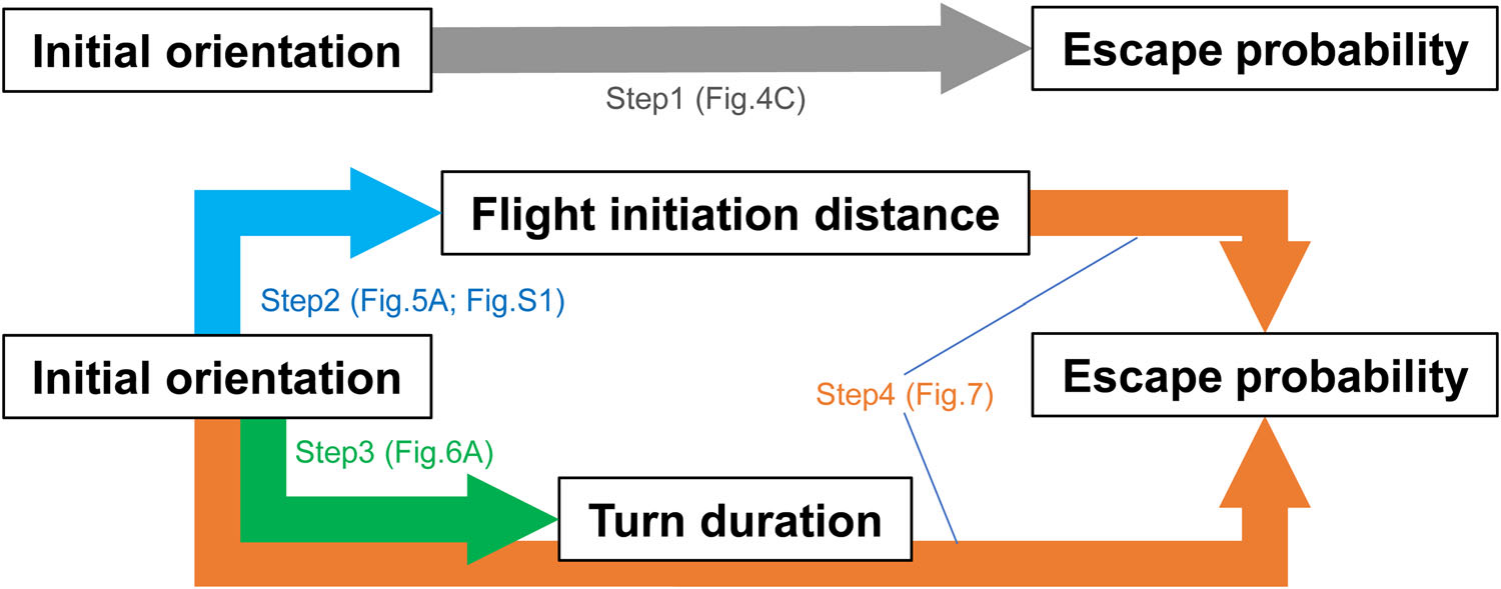
**Schematic of the two possible pathways in which initial orientation affects escape probability.**

The C-start escape response of fish and amphibian larvae is one of the most well-studied escape responses in animals (Domenici and Blake, 1997; Eaton et al., 2001). The C-start escape response is composed of three distinct stages based on kinematics: the initial bend (stage 1), the return tail flip (stage 2) and then continuous swimming or coasting (stage 3) (Domenici and Blake, 1997; Weihs, 1973). Flight initiation distance, escape speed, turning speed and escape trajectory affect evasion outcome (Scharf et al., 2003; Stewart et al., 2013; Walker et al., 2005; Webb, 1982; Webb and Zhang, 1994). In addition, the initial orientation does not affect evasion outcome in zebrafish larvae evading from adult zebrafish (Stewart et al., 2013), however, we believe this study is insufficient for the following reason: Domenici and Blake (1993b) hypothesized that the optimal initial orientation of prey should occur at an intermediate value (i.e. 130° away from predators) by balancing two conflicting demands: minimizing the time required to turn away and keeping its predator within its visual perception range. Considering this hypothesis, the initial orientation would affect escape probability through two different pathways: changes in responsive variables (e.g. responsiveness, flight initiation distance) and turn duration. However, Stewart et al. (2013) examined the effect of initial orientation on escape probability separately from these variables. Therefore, the objectives of our study were to determine whether initial orientation affects evasion outcome and, if so, to examine the above possible pathways. To achieve these objectives, we recorded the escape responses of juvenile red sea bream [*Pagrus major* (Temminck and Schlegel, 1843)] to the predatory scorpion fish [*Sebastiscus marmoratus* (Cuvier, 1829)], and analyzed the data in four steps (Fig. 1). (1) We examined whether the prey fish showed maximum escape probability at an intermediate initial orientation value. (2) By examining the relationship between the initial orientation and responsive variables, we tested whether the prey fish had spatial bias in detecting the attacking predator. (3) We tested whether an increase in the initial orientation of prey fish (more opposite from the direction of the predator) decreased turn duration. (4) Because the turn duration could not be calculated for the captured individuals and there was a clear linear relationship between initial orientation and turn duration, we modeled the effects of flight initiation distance and initial orientation on escape probability to verify the existence of two pathways.

## RESULTS

**Fig. 2.**
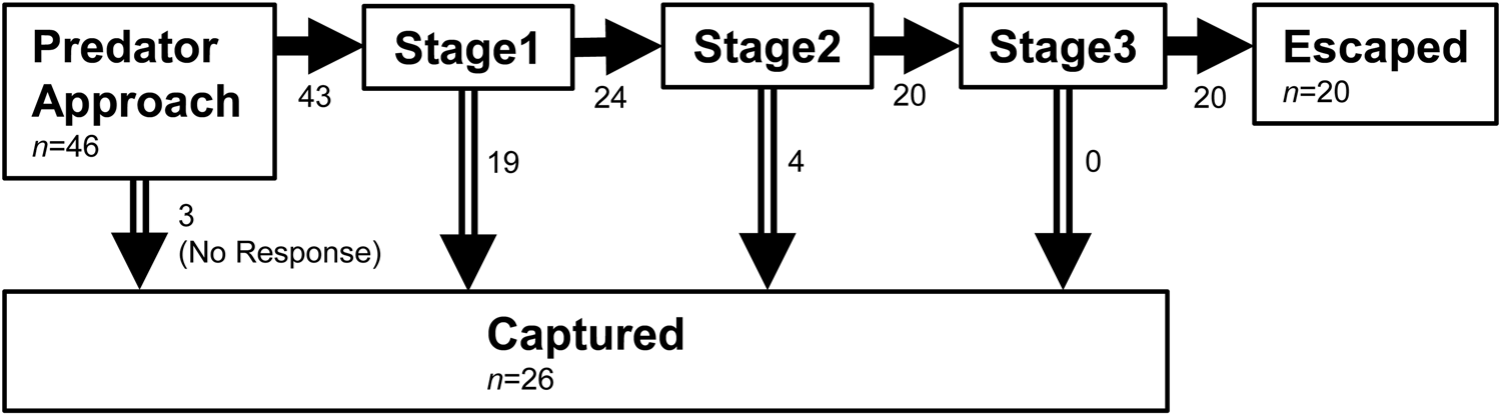
**Diagram showing the kinematic stages in which the prey were captured.**

In general, the predator [*S. marmoratus*, 149.9±17.0 mm (mean±s.d.) total length (TL), *n*=7] approached the prey (*P. major*, 56.1±9.6 mm TL, *n*=46) and then attacked it by opening its mouth. The kinematic stages in which the prey were captured are summarized in Fig. 2. Most prey individuals (93%) showed escape responses (C-start), but three (7%) did not show responses and were captured by predators. Of the 43 prey that showed escape responses, 19 (44%) were captured by predators during stage 1. Of the 24 prey that survived until the end of the stage 1, four (17%) were captured by the end of stage 2. No fish were captured during stage 3. Of the total number of prey captured (26), 22 (85%) were captured by the end of stage 1. These results indicate that stage 1 is the most critical period for *P. major* to escape from the attack of *S. marmoratus*.

**Fig. 3.**
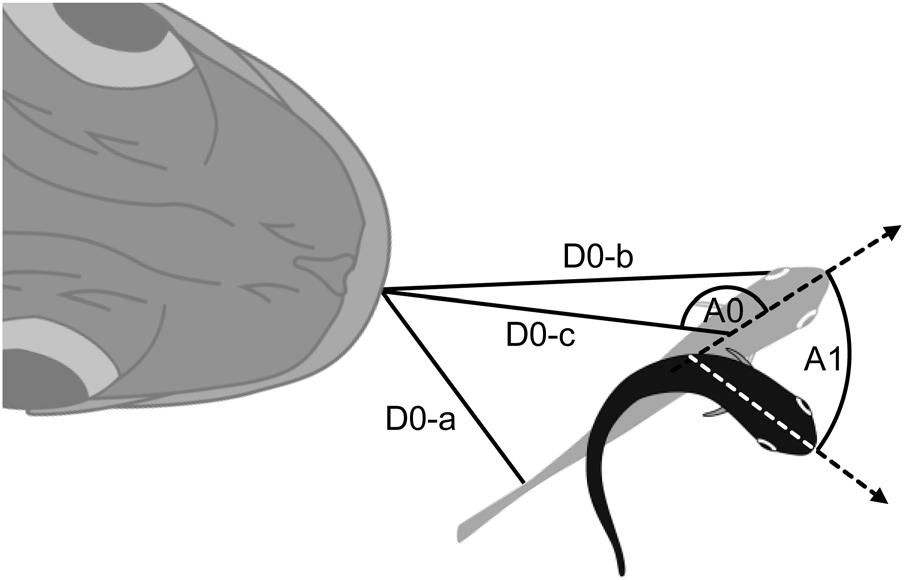
**Schematic drawing of measured variables.** The position of the prey at the onset of the escape response is shown as a gray fish and the position at the end of stage 1 is shown as a black fish. D0-a, flight initiation distance calculated using the closest margin of the prey's body to the predator's snout (FID_body_); D0-b, flight initiation distance calculated using the nearer prey's eye (FID_eye_); D0-c, flight initiation distance calculated using the prey's center of mass (FID_CM_); A0, initial orientation; A1, turn angle.

**Fig. 4.**
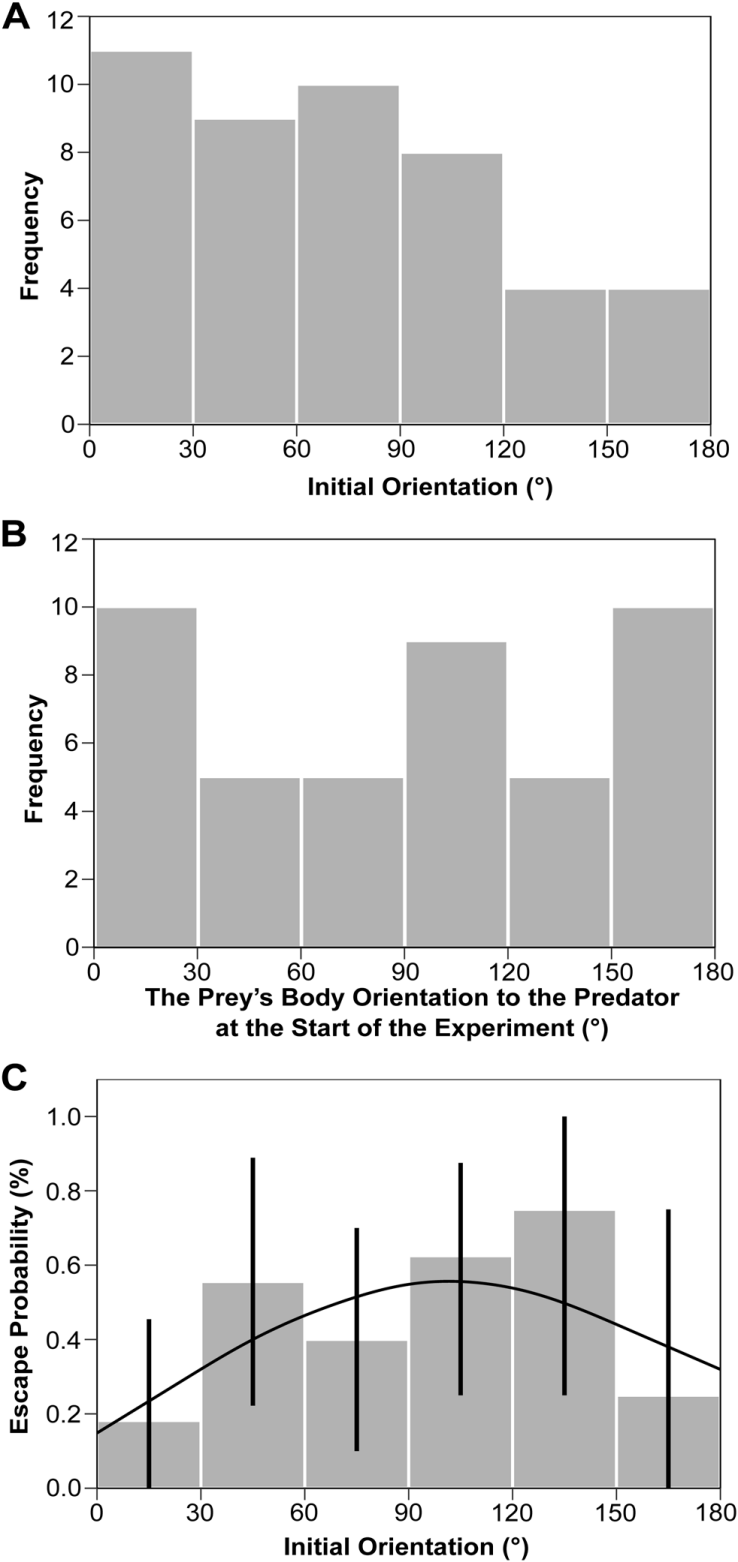
(A) Frequency distribution of initial orientations (*n*=46). (B) Frequency distribution of the prey's body orientation to the predator at the start of the experiment (*n*=44). (C) Relationship between initial orientation and escape probability. The error bars represent 95% confidence intervals, estimated by assuming binomial distributions. The line was estimated by mixed effects logistic regression analysis (*n*=46, *χ^2^*=4.32, *P*=0.12).

The frequency distribution of the initial orientation (i.e. the prey's body orientation to the predator's snout at the onset of escape response; Fig. 3, A0), the frequency distribution of the prey's body orientation to the predator at the start of the experiment (i.e. the prey's body orientation to the predator's snout when the acclimation pipe for the prey was removed) and the initial orientation–escape probability relationship are shown in Fig. 4A, B and C, respectively. The frequency of initial orientation at 120-180° was lower than at 0-120° (Fig. 4A), whereas the frequency of the orientation at the start of the experiment at 120-180° was similar to that at 0-120° (Fig. 4B). Escape probability was the highest in the 120-150° initial orientation bin, although 95% confidence intervals based on binomial distributions suggest that there were no significant differences among the different initial orientation bins (Fig. 4C). The peak in escape probability occurred at 94.7° in the logistic regression curve (Fig. 4C), although this tendency was not statistically significant [Likelihood Ratio (LR) test, *χ²*=4.32, d.f.=2, *P*=0.12].

**Fig. 5.**
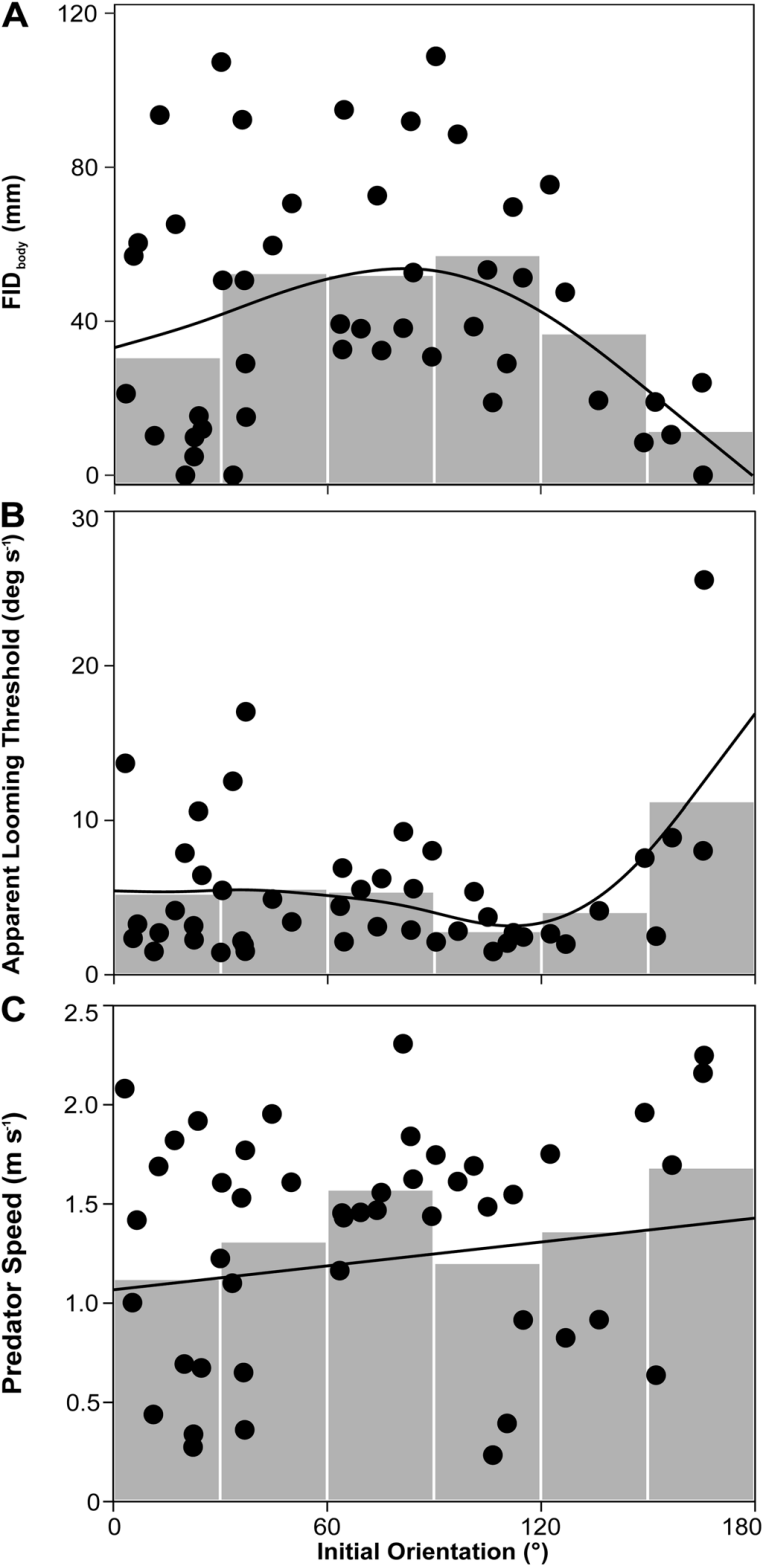
(A) Relationship between initial orientation and flight initiation distance calculated using the closest margin of the prey's body to the predator's snout (FID_body_). The line was estimated by the generalized additive mixed model (GAMM; *F*=2.56, *P*=0.11), in which the predator speed was regarded as its mean value (1.42 m s^−1^). (B) Relationship between initial orientation and apparent looming threshold (ALT). The line was estimated by the GAMM (*F*=2.94, *P*<0.05). (C) Relationship between initial orientation and predator speed. (GAMM, *F*=1.59, *P*=0.21). All the prey fish were used in these analyses (*n*=46). The grey bars represent the mean values for the 30° initial orientation bins.

There was no observable pattern in the initial orientations of the three prey individuals that did not show escape responses (19.9, 33.4 and 165.7°). Flight initiation distance calculated using the closest margin of the prey's body to the predator's snout (FID_body_; Fig. 3, D0-a) was the shortest when the initial orientation was away from predators (150-180°) and the second shortest when the initial orientation was toward predators (0-30°; Fig. 5A), although this tendency (the effect of initial orientation on FID_body_) was not statistically significant [generalized additive mixed model (GAMM), *F*=2.56, estimated d.f.=2.28, estimated residual d.f.=40.30, *P*=0.11]. Predator speed significantly increased the FID_body_ (GAMM, *F*=5.76, estimated d.f.=2.41, estimated residual d.f.=40.30, *P*<0.05). The relationships between initial orientation and the other two flight initiation distances calculated using the prey's center of mass and the nearer prey's eye (FID_eye_ and FID_CM_; Fig. 3, D0-b, D0-c) were similar to the initial orientation-FID_body_ relationship (Fig. S1). The apparent looming threshold (ALT) at which the prey responds to the predator's strike, measured by the rate of change of the predator's frontal profile as viewed by the prey (Dill, 1974; Webb, 1982, 1986), was the largest when the initial orientation was away from predators (150-180°) and the values were similar among the other initial orientations (0-150°; Fig. 5B); this tendency (the effect of initial orientation on ALT) was statistically significant (GAMM, *F*=2.94, estimated d.f.=3.54, estimated residual d.f.=41.46, *P*<0.05). Predator speed was the highest when the initial orientation was toward predators (0-30°; Fig. 5C), although this tendency (the effect of initial orientation on predator speed) was not statistically significant (GAMM, *F*=1.59, estimated d.f.=1, estimated residual d.f.=44, *P*=0.21).

**Fig. 6.**
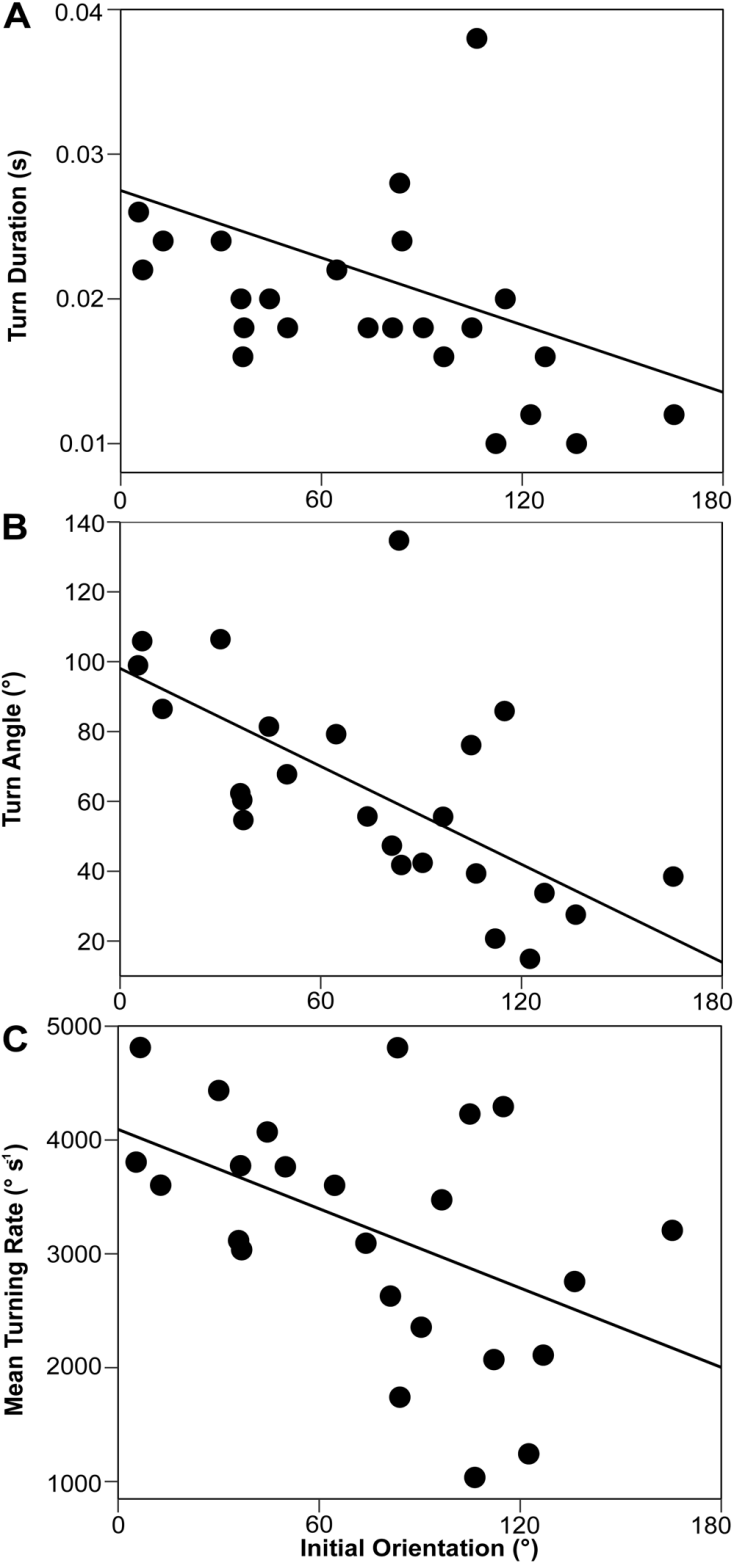
(A) Relationship between initial orientation and turn duration (*R*= −0.41, *P*<0.05). (B) Relationship between initial orientation and turn angle (*R*= −0.61, *P*<0.01). (C) Relationship between initial orientation and mean turning rate (*R*= −0.48, *P*<0.05). Prey fish that survived until the end of stage 1 were used in A, B and C (*n*=24).

The relationship between initial orientation and prey kinematic variables are summarized in Table 1. There were negative relationships between initial orientation and turn angle (Fig. 6A; *R*= −0.61, *n=*24, *P*<0.01), between initial orientation and turn duration (Fig. 6B; *R*= −0.41, *n*=24, *P*<0.05), and between initial orientation and mean turning rate (*R*= −0.48, *n=*24, *P*<0.05). There was a positive relationship between initial orientation and cumulative distance (*R*=0.45, *n=*26, *P*<0.05). There were no significant relationships between initial orientation and the other variables (Table 1; Fig. S2). Additionally, there was a significant positive relationship between turn angle and turn duration (*R*=0.53, *n*=24, *P*<0.01), but there was no significant relationship between mean turning rate and turn duration (*R*=0.10, *n=*24, *P*=0.64).

**Table 1.**
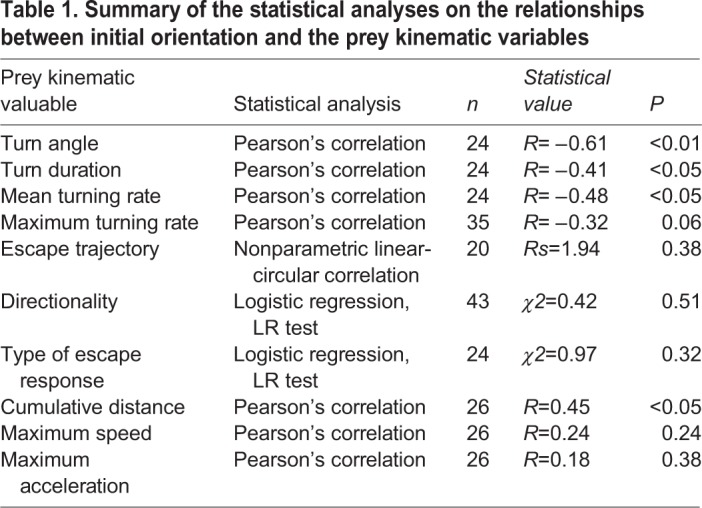
**Summary of the statistical analyses on the relationships between initial orientation and the prey kinematic variables**

**Fig. 7.**
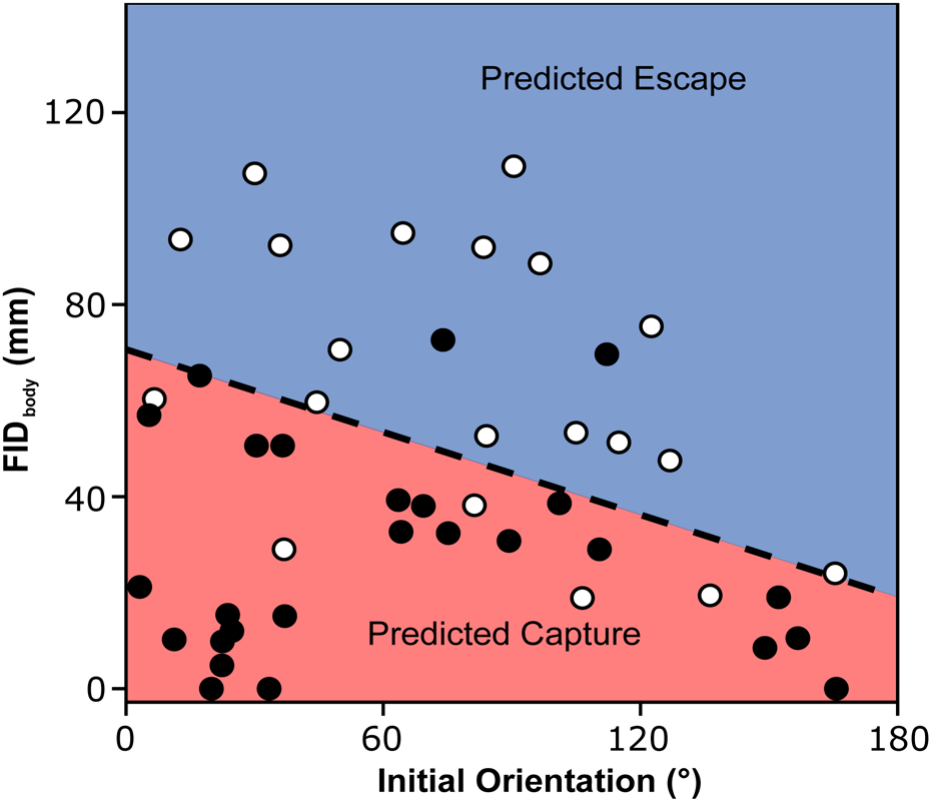
**The combination of initial orientation and flight initiation distance calculated using the closest margin of the prey's body to the predator's snout (FID_body_) for predicting the outcomes of predator-prey interactions (effect of FID_body_, *χ^2^*=8.50, *P*<0.01; effect of initial orientation, *χ^2^*=4.41, *P*<0.05).** Open circles are indicative of successful escape from a predator's attack and filled circles are indicative of prey captured by a predator's attack. The dashed line represents the 50% escape probability estimated from the mixed effects logistic regression analysis, and the blue and red areas represent the predicted escape and capture, respectively. Of the 46 data points used in the analysis, 39 (84.8%) were correctly categorized by the estimated line.

Differences in the parameters between the successful (escaped) and unsuccessful (captured) escapes are shown in Table 2. The smallest Akaike's information criterion (AIC) was obtained for the model composed of the effects of FID_body_ and initial orientation (Table 3). FID_body_ of the successful escapes (63.9±29.3 mm) was larger than that of the unsuccessful ones (28.2±22.2 mm), and increases in FID_body_ significantly increased escape probability (Fig. 7; LR test, *χ^2^*=20.72, d.f.=1, *P*<0.01). The odds ratio of FID_body_ indicates that an increase of 30.9 mm (1 s.d.) increased the escape probability 7.39 times. The initial orientation of the successful escapes (79.7±43.5°) was larger than that of the unsuccessful ones (64.2±51.0°) and when the effect of FID_body_ was offset, the larger initial orientation significantly increased escape probability (Fig. 7; LR test, *χ^2^*=4.41, d.f.=1, *P*<0.05). The odds ratio indicates that a 48.0° (1 s.d.) increase in initial orientation increased the escape probability 2.44 times. FID_eye_ and FID_CM_ of the successful escapes (68.8±27.4 and 72.9±30.0 mm) were larger than those of the unsuccessful ones (33.5±22.9 and 39.3±24.6 mm), as was the case with FID_body_. The other variables of successful escapes were similar to those of the unsuccessful ones (Table 2).

**Table 2.**
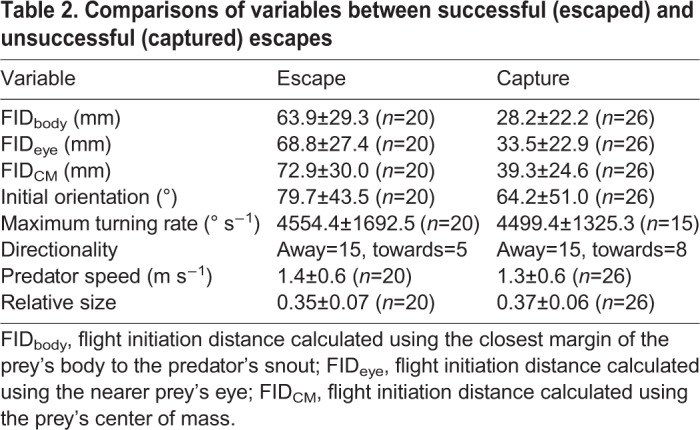
**Comparisons of variables between successful (escaped) and unsuccessful (captured) escapes**

**Table 3.**
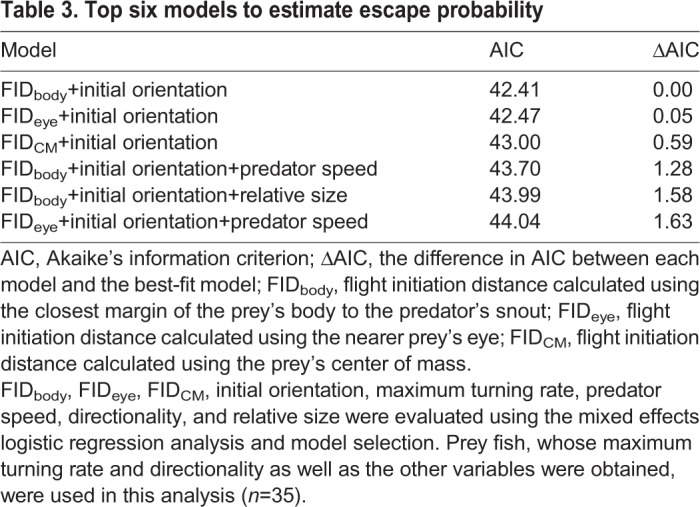
**Top six models to estimate escape probability**

## DISCUSSION

Although the probability of *P. major* juveniles escaping from the predatory strikes of *S. marmoratus* was the highest in the 120-150° initial orientation bin and its peak occurred at 94.7° in the logistic regression curve (Fig. 4C), the effect of initial orientation on the escape probability was statistically insignificant. However, this statistical insignificance would be attributed to the small sample size, specifically at 150-180° initial orientation (*n*=4; Fig. 4A), because when 20 datasets (initial orientation and escape outcome) were randomly sampled with replacement in each 30° initial orientation bin to conduct generalized linear mixed model (GLMM) analysis and this process was repeated 1000 times, the effect of initial orientation became significant in 994 cases (LR test, median *χ^2^*=124.64, d.f.=2, median *P*<0.01). Additionally, the large variation in the relationship between initial orientation and flight initiation distance (Fig. 5A; Fig. S1) might have masked the clear relationship between initial orientation and escape probability. Given these facts, it is likely that the escape probability was actually the highest at an intermediate initial orientation value; however, we acknowledge that further research with a larger sample size, specifically at large initial orientations, is required to confirm this hypothesis.

Our results show that initial orientation affects the escape probability through two different pathways. The first pathway is through the flight initiation distance. When the initial orientation was away from predators (150-180°), flight initiation distance (either FID_body_, FID_eye_ or FID_CM_) was the shortest. This may be related to a sensory perception range in the prey, as discussed in Seamone et al. (2014). The C-start escape response is triggered by either visual (Dill, 1974; Dunn et al., 2016) or mechanical stimuli (Stewart et al., 2013, 2014; Umeda et al., 2016). When it is triggered by visual stimuli, a blind zone would exist for prey in the rear (Tisdale and Fernández-Juricic, 2009; Tyrrell and Fernandez-Juricic, 2015). Indeed, the prey species *P. major* has a visual blind zone in the 160-180° initial orientation (Kawamura, 2000) and the ALT, the rate of change of the predator's frontal profile at the onset of the escape response, was larger when attacked from behind (150-180° initial orientation) than when attacked laterally or head on (0-150° initial orientation) (Fig. 5B). However, the lateral line (mechanosensory system) is distributed throughout the body (Dijkgraaf, 1963; Kasumyan, 2003), which may allow 360° perception without any spatial bias. Thus, the short flight initiation distance and large ALT at large initial orientation could be attributed to the fact that the prey could not see the predator approaching from behind and responded via mechanosensory system. An alternative explanation is that the predator's frontal profile entered the visual field of the prey at some point even when attacked from behind (150-180° initial orientation), which allowed the prey to finally respond to the predator via visual sense. Further research is needed to clarify the sensory mechanisms involved in the short flight initiation distance in the large initial orientation.

The flight initiation distance (either FID_body_, FID_eye_ or FID_CM_) was the second shortest when the initial orientation was toward predators (0-30°; Fig. 5A; Fig. S1). However, this would be attributable to the slow speed of the predator in the 0-30° initial orientation (Fig. 5C). When a predator speed is smaller, the rate of change of a predator's frontal profile and the bow wave of a predator, both of which can trigger prey escape response, are smaller for prey fish (Dill, 1974; Stewart et al., 2014). In fact, the ALT, a combined variable between the flight initiation distance and predator speed, when attacked head on (0-30° initial orientation) was similar to that when laterally attacked (30-150° initial orientation) (Fig. 5B). Therefore, it is likely that the prey fish have no spatial bias in detecting an attacking predator except for a blind zone in the rear.

The second pathway in which the initial orientation affects escape probability is through turn duration. Our results show that an increase in the initial orientation decreases turn duration, turn angle and mean turning rate (Fig. 6). There was a significant positive relationship between turn angle and turn duration, but there was no significant relationship between mean turning rate and turn duration. Therefore, it is likely that the initial orientation-mediated turning rate change has a relatively minor effect on the turn duration, and the observed initial orientation–turn duration relationship is mainly attributed to the change in turn angle. The initial orientation–turn angle and turn angle–turn duration relationships are consistent with studies of many animal taxa (e.g. other fish, frogs, cockroaches and lizards) (Camhi and Tom, 1978; Cooper and Sherbrooke, 2016; Domenici and Batty, 1994, 1997; Domenici and Blake, 1991; Domenici et al., 2004; Eaton and Emberley, 1991; Ellerby and Altringham, 2001; King and Comer, 1996). C-starts and other escape responses start from initial turns, followed by escape locomotion; during the initial turns, the animals do not move large distances but stay close to their initial positions (Camhi et al., 1978; Domenici and Blake, 1997; King and Comer, 1996; Tauber and Camhi, 1995). Thus, predators are able to approach the prey during these initial turns. In fact, our results show that an increase in the initial orientation increases the cumulative distance that the prey traverse within a set time period (Fig. S2). It is thus highly likely that initial orientation-mediated turn duration changes affect escape probability by changing the time available for the predator to approach the prey before the initiation of escape locomotion.

Our results show that when the effect of flight initiation distance is offset, an increase in the initial orientation (i.e. more fully away from the predator) linearly increases escape probability (Fig. 7). This is most likely because the non-linear effect of initial orientation on escape probability is mainly attributed to the pathway through flight initiation distance, and after removing its effect, the remaining effect of initial orientation occurs solely though turn duration which linearly affects escape probability. This idea was overlooked in a study of zebrafish larvae evading adult zebrafish (Stewart et al., 2013) in which escape probabilities were compared only among six different initial orientation bins. In fact, the relationships between initial orientation and flight initiation distance, and between initial orientation and escape probability observed in their study are similar to those in our study, in that escape probability was smallest in the smallest initial orientation and second smallest in the largest initial orientation, and flight initiation distance was shortest in the largest initial orientation and second shortest in the smallest initial orientation (Figs 4 and 6 in Stewart et al., 2013). Therefore, although our study has a smaller sample size (*n*=46, especially small at large initial orientations) compared to the study on zebrafish larvae (*n*=66) and thus the statistical analysis should be considered with care, the initial orientation may actually be a crucial parameter for predator evasion in other fish as well.

Our results, that the maximum escape probability occurred at the 120-150° initial orientation bin and its peak occurred at 94.7° in the logistic regression curve (Fig. 4C), together with the results on the effects of initial orientation on the flight initiation distance (Fig. 5A; Fig. S1) and turn duration (Fig. 6A) and combined effect of initial orientation and flight initiation distance on escape probability (Fig. 7), support the Domenici and Blake hypothesis that optimal initial orientation of prey should be an intermediate value by balancing two conflicting demands: minimizing the time for turning away and keeping the predator within visual perception range (Domenici and Blake, 1993b). However, the frequency of the initial orientation was not highest around this range: the frequency at 120-180° was smaller than that at 0-120° (Fig. 4A). Because we used naïve hatchery-reared fish that had not experienced any predators, the prey might not have recognized the predator as dangerous, and thus the prey did not adjust the initial orientation in advance. Black goby change their posture when a weak stimulus is presented before the strong stimulation that finally elicits an escape response (Turesson et al., 2009). Therefore, prey animals that recognize a predator in advance may adjust their initial orientation to maximize their escape probability. Alternatively, the predators could have adjusted their attack angle (i.e. initial orientation) to the front to maximize predation probability because the frequency of the prey's body orientation to the predator at the beginning of the experiment at 120-180° was similar to that at 0-120° (Fig. 4B), and because we used wild *S. marmoratus* as predators.

Different geometrical models have been proposed to explain the factors affecting escape probability and/or the escape trajectory (Arnott et al., 1999; Corcoran and Conner, 2016; Domenici, 2002; Howland, 1974; Weihs and Webb, 1984), but none of these models have incorporated initial orientation. Furthermore, initial orientation has not been considered in many empirical studies of predator-prey interactions (e.g. Dangles et al., 2006; Fuiman, 1993; Scharf et al., 2003; Walker et al., 2005). Our results clearly show that initial orientation affects escape probability through two pathways: changes in flight initiation distance and turn duration. These findings highlight the importance of incorporating data on initial orientation and its related variables into both theoretical and empirical studies of predator-prey interactions.

## MATERIALS AND METHODS

### Ethics statement

Animal care and experimental procedures were approved by the Animal Care and Use Committee of the Institute for East China Sea Research, Nagasaki University (Permit No. ECSER15-12), in accordance with the Regulations of the Animal Care and Use Committee of Nagasaki University.

### Fish samples

Hatchery-reared *P. major* (*n*=151) were utilized as prey fish in this study. All individual *P. major* were provided from commercial hatcheries, and were kept in three 200 l polycarbonate tanks at the Institute for East China Sea Research, Nagasaki University, Japan. They were fed with commercial pellets (Otohime C2; Marubeni Nisshin Feed Co. Ltd, Tokyo, Japan) twice a day.

As predators, we used *S. marmoratus* (*n*=7), which is a common reef predator around the coast of Japan. *S. marmoratus* usually employs a ‘stalk-and-attack’ tactic. All *S. marmoratus* were collected by hook-and-line around Nagasaki prefecture, Japan. The collected *S. marmoratus* were kept in a glass aquarium (1200×450×450 mm) before the start of the experiment. They were standardly fed krill once every 2-4 days.

The position of the center of mass (CM) for *P. major* was estimated by hanging dead fish (54.3±3.3 mm TL, *n*=10) from two different points using a suture and needle (Lefrancois et al., 2005). The CM position from the tip of the head was estimated as 34±1% of the TL.

### Experimental procedure

Experiments were performed in a glass aquarium (900×600×300 mm) filled with seawater to a depth of 100 mm. The water temperature during the experiments was 23.1±0.9°C. White plastic plates with grid lines were placed on the bottom and three sides of the tank; one side (900×300 mm) of the tank was left transparent to record the side view of the fish. A preliminary experiment showed that *S. marmoratus* actively fed in low light conditions, so two LED bulbs covered with red cellophane were used to illuminate the tank. The light intensity was maintained at 54 lux. Two synchronized high-speed video cameras (HAS-L1; Ditect Co., Tokyo, Japan) were used to record dorsal and side views of the fish simultaneously (note that we only used the dorsal views in this study).

An individual *S. marmoratus* starved for at least 24 h was first introduced into the experimental tank and allowed to acclimate for 30 min. An individual *P. major* was then introduced into a PVC pipe (60 mm diameter) with 112 small holes (3 mm diameter), set in the center of the tank, and acclimated for 15 min. The 15 min period was chosen because a preliminary experiment showed that the fish settled down and opercular beat frequency recovered to the basal level within, at most, 15 min. After the acclimation period, the trial was started by slowly removing the PVC pipe to release the *P. major*. When *S. marmoratus* attacked the *P. major*, we recorded the movements of both predator and prey using the high-speed video cameras. If *S. marmoratus* did not show any predatory movements for 20 min, the trial was ended. Seven *S. marmoratus* were repeatedly used, but each *P. major* was used only once.

### Analysis of video sequences

Because the vertical displacements of both fish were negligible, we only used the dorsal video views in our analyses. Before measuring the kinematic and behavioral variables, we noted the kinematic stage in which each prey was captured. In a few cases, the predator grabbed or touched the prey, but the prey finally escaped from the predator. Because this study focused on sensory capabilities and kinematic performance rather than the other defensive tactics (e.g. size, spines), these cases were regarded as captured. The escape response of *P. major* and the predatory strike of *S. marmoratus* were then analyzed frame by frame using Dipp-Motion Pro 2D (Ditect Co.). The CM, the tip of the snout, and the eye positions of *P. major* and the tip of the snout of *S. marmoratus* were digitized in each frame. The closest margin of the prey's body to the predator's snout was digitized in the frame at the onset of stage 1. The following variables were then calculated from these points.

We calculated three different flight initiation distances: FID_body_, the distance between the predator's snout and the closest margin of the prey's body at the onset of stage 1 (Fig. 3, D0-a) (Stewart et al., 2013); FID_eye_, the distance between the predator's snout and the nearer prey's eye at the onset of stage 1 (Fig. 3, D0-b) (Meager et al., 2006); and FID_CM_, the distance between the predator's snout and the prey's CM at the onset of stage 1 (Fig. 3, D0-c) (Seamone et al., 2014; Walker et al., 2005). FID_body_ was calculated because the escape response can be triggered by mechanical stimuli (Stewart et al., 2013, 2014) and the lateral line (mechanosensory system) is distributed throughout the body (Dijkgraaf, 1963; Kasumyan, 2003). Additionally, many predators could catch a prey by grabbing any part of the body, and thus FID_body_ would also provide an ecological explanation. FID_eye_ was calculated for providing a sensory explanation because the escape response can be triggered by visual stimuli (Dill, 1974; Dunn et al., 2016). FID_CM_ was calculated because this flight initiation distance had previously been used (Seamone et al., 2014; Walker et al., 2005) and a predator *Micropterus salmoides* tends to attack the prey's CM as the target (Webb, 1986). Indeed, the mean strike target of the predator *S. marmoratus* on the stationary prey *P. major* [i.e., the intersection point between the predator's strike path (calculated as the regression line of the predator's snout during the period between the onset of the mouth opening and 0.02 s before the onset of the mouth opening) and the prey's body midline at the onset of the predator's mouth opening] was 33±29% of the prey's TL (±95% confidence interval, *n*=18), which was nearly equivalent to the prey's CM (34% of the TL).

The other variables are calculated as follows. Initial orientation (°): the angle between the line passing through the predator's snout and the prey's CM, and the line passing through the prey's CM and the prey's snout at the onset of the stage 1 (Fig. 3, A0). Thus, a small initial orientation means that the prey fish is attacked head on and a large initial orientation means that the prey fish is attacked from behind. Turn angle (°): the angle between the line passing through the prey's CM and the prey's snout at the onset of stage 1, and the line passing through the prey's CM and the prey's snout at the onset of the return tail flip (Fig. 3, A1). Turn duration (s): the time between the onset of stage 1 and the onset of the return tail flip. Mean turning rate (° s^−1^): the turn angle divided by the turn duration. Maximum turning rate (° s^−1^): the maximum angular velocity within the turn duration. Escape trajectory (°): the angle between the line passing through the prey's CM and the predator's snout at the onset of stage 1, and the line passing through the prey's CM and the prey's snout at the end of the return tail flip. Directionality (away or towards response): away response was defined as the response in which the first detectable movement was oriented away from the predator and towards response was defined as the response in which the first detectable movement was oriented towards the predator. Type of escape response (double or single bend): double bend response was defined as the response that had a contralateral muscle contraction after the initial turn (stage 1) and single bend response was defined as the response that lacked a contralateral muscle contraction after the initial turn. Predator speed (m s^−1^): the cumulative distance the predator's snout moves during the period between the onset of stage 1 and 0.02 s before the onset of stage 1, multiplied by 50. ALT (° s^−1^): the threshold at which the prey responds to the predator's strike, measured by the rate of change of the predator's frontal profile as viewed by the prey (Dill, 1974; Webb, 1982, 1986). ALT was calculated as (4 *US*)/(4*D*^2^+*S*^2^), where *U* is the predator speed (see above for details), *S* is the predator's frontal profile calculated as the mean of maximal depth and maximal width and *D* is the sum of the distance between the nearer prey's eye position and the predator's snout, and the distance between the predator's snout and the point where the predator's maximal depth and maximal width is located. These morphological features of the predator were measured in each specimen (*n*=7) to the nearest 0.01 mm using a digital caliper at the end of the experiment.

The time-distance variables [cumulative distance (mm), maximum speed (m s^−1^) and maximum acceleration (m s^−2^)] were measured based on the displacement of the CM. The variables were evaluated within a fixed 0.02 s duration. The 0.02 s duration was chosen because all captured fish were captured before the end of stage 2 (Fig. 2), the average duration for stage 1 and 2 was 0.02 s and the peak speed and acceleration usually occurred before stage 2 ended (Domenici, 2009). Speed and acceleration were calculated by first- and second-order differentiation, respectively, of the cumulative distance for the time-series. A Lanczos five-point quadratic moving regression method (Walker, 1998) was applied to calculate these values using custom R program (The R Foundation for Statistical Computing, Vienna, Austria).

When prey fish did not show escape responses (*n*=3, Fig. 2), FID_body_, FID_eye_ and FID_CM_ were regarded as 0. The initial orientation relative to a predator was calculated at the onset of the predator's strike. The predator speed was calculated during the period between the time of capture and 0.02 s before the time of capture.

Of the total number of prey captured (26), 22 (85%) were captured by the end of stage 1 (Fig. 2) and thus, many prey kinematic variables (i.e. turn angle, turn duration, mean turning rate, escape trajectory, type of escape response and time-distance variables) could not be calculated for most of the captured individuals. Accordingly, these variables were not incorporated in the analysis to examine the factors affecting the escape probability (see ‘Statistical analyses’ section for details). Maximum turning rate was calculated in many of the captured individuals (58%) because it occurred around the middle of stage 1 (Domenici and Blake, 1991).

### Statistical analyses

Of the 151 digital films recorded, 46 were used for data analyses. First, fish that were not sufficiently far from the wall (more than one TL) were omitted from the analysis to eliminate possible wall effects (Eaton and Emberley, 1991). Second, only fish that initiated an escape response from a state of rest were used in the analysis (we excluded cases where *S. marmoratus* chased *P. major* that were already swimming).

To examine whether the optimal initial orientation to escape predators occurred at an intermediate value, we looked for the peak in escape probability using a mixed effects logistic regression analysis (generalized linear mixed model with a binomial error distribution and a logit link function) (Zuur et al., 2009). Success and failure of predator evasion were designated as 1 and 0, respectively, and were used as the objective variable. Initial orientation and its square were used as the explanatory variables, because escape probability is likely to change in response to changes in initial orientation in a non-linear fashion because of two conflicting demands: minimizing the time for turning away and keeping the predator within its visual perception range (Domenici and Blake, 1993b). All the fish were used in this analysis (*n*=46). Predator ID was included as a random factor because unknown predator abilities may have affected the evasion outcome. The significance of the explanatory variables was then assessed by removing them from the model and comparing the change in deviance using the LR test with a χ^2^ distribution.

Prey animals can have spatial bias in detecting an attacking predator (e.g. from a sensory blind zone) (Domenici, 2002; Tisdale and Fernández-Juricic, 2009; Tyrrell and Fernandez-Juricic, 2015). Therefore, we examined whether the initial orientation affected the responsive variables. Because a majority of the prey (93%) showed escape responses, we could not conduct any statistical analysis regarding responsiveness. Instead, we examined whether initial orientation affected the flight initiation distance (FID_body_, FID_eye_ or FID_CM_) and ALT. We separately examined these variables because flight initiation distance directly affects escape probability as it determines the time for a predator to reach prey animals (Walker et al., 2005) and ALT explains the mechanism of how prey animals respond to an attacking predator (Dill, 1974). Additionally, to explore the mechanism in the observed relationships between initial orientation and flight initiation distance or ALT, the effect of initial orientation on predator speed was also examined. The GAMM with a normal error distribution and an identity link function (Zuur et al., 2009) was used for the analysis, because flight initiation distance (and possibly ALT and predator speed) is likely to change in response to changes in initial orientation in a non-linear fashion (Tisdale and Fernández-Juricic, 2009; Tyrrell and Fernandez-Juricic, 2015). All the fish were used in this analysis (*n*=46). Flight initiation distance, ALT and predator speed were used as the objective variables, and initial orientation was considered as an explanatory variable. Predator ID was also included as a random factor. In the analysis to estimate the flight initiation distance, the predator speed was also incorporated as covariate because predator speed can also change the flight initiation distance (Stewart et al., 2013). The significance of the explanatory variables was assessed by the *F*-test.

We tested whether an increase in the initial orientation of prey fish (more opposite from the direction of the predator) decreased turn duration using a Pearson's correlation coefficient. Because the other prey kinematic variables can be affected by initial orientation, we also examined their relationships with initial orientation using the following methods. The turn angle, mean turning rate, maximum turning rate, cumulative distance, maximum speed and maximum acceleration were examined using a Pearson's correlation coefficient. Because the escape trajectory is a circular variable and is unlikely to have a linear relationship with initial orientation (Domenici et al., 2011a,b), a nonparametric linear-circular correlation coefficient was used to test the relationship. The type of escape response (double bend or single bend) may have changed in response to the initial orientation, as more single bend responses with smaller initial orientations and more double bend responses with larger initial orientations (Domenici and Blake, 1993a) and thus was analyzed using a logistic regression analysis. Single bend and double bend responses were designated as 0 and 1, respectively, and were used as the objective variable, while initial orientation was used as the explanatory variable. The significance of the initial orientation was then assessed by removing it from the model and comparing the change in deviance using the LR test. The directionality (away or towards response) may have changed in response to the initial orientation, as more towards responses with the smallest and largest initial orientations and more away responses with intermediate values in initial orientations (Domenici and Blake, 1993b), and thus was analyzed using a logistic regression analysis (Zuur et al., 2009). Towards and away responses were designated as 0 and 1, respectively, and were used as the objective variable, while initial orientation and its square were used as the explanatory variables. The significance of the explanatory variables was then assessed by removing them from the model and comparing the change in deviance using the LR test.

To test the hypothesis that initial orientation-mediated changes in flight initiation distance and turn duration affect escape probability, we modeled the effect of flight initiation distance (either FID_body_, FID_eye_ or FID_CM_) and initial orientation on escape probability (Fig. 1). We incorporated initial orientation instead of turn duration because turn duration could not be calculated for most of the captured individuals and there was a clear linear relationship between initial orientation and turn duration. In other words, the effect of initial orientation was examined on condition that the pathway through flight initiation was offset. The effects of flight initiation distance and initial orientation on escape probability were evaluated using a mixed effects logistic regression analysis and model selection (Zuur et al., 2009). Success and failure of predator evasion were designated as 1 and 0, respectively, and used as the objective variable. Initial orientation and flight initiation distance were considered as explanatory variables. Maximum turning rate, directionality, predator speed, and relative size of prey to predator (prey's TL divided by predator's TL) were also included in the model as covariates because these variables significantly affected escape probability in previous studies (Catania, 2009; Dangles et al., 2006; Scharf et al., 2003; Stewart et al., 2013; Walker et al., 2005). Although the other prey kinematic variables (i.e. cumulative distance, maximum speed, maximum acceleration, mean turning rate, escape trajectory and type of C-start) could affect escape probability (Domenici, 2009; Walker et al., 2005), we could not incorporate them into the analysis because most of the captured fish (85%) were captured before the end of stage 1 and thus the data points of these individuals were not enough to calculate these variables. Predator ID was included as a random factor because unknown predator abilities may affect the evasion outcome. Prior to the model selection, relationships between all pairs of continuous explanatory variables (except for directionality, which is a binary variable) were examined using a Pearson's correlation coefficient. Because FID_body_, FID_eye_ and FID_CM_ were highly correlated with each other (Table S1), and because we had no prior knowledge on which flight initiation distance best predicted escape probability, sets of candidate models were constructed using each flight initiation distance. A total of 128 candidate models were constructed, and AIC was used to select the most parsimonious model. To further verify the effects of selected variables on escape probability, the significance of the variables was assessed by progressively removing them from the best-fit model and comparing the change in deviance using the LR test. Because sample sizes of the maximum turning rate and directionality were limited to 35 and 43, respectively, the model selection analysis was performed using 35 datasets. The LR test was performed using all 46 datasets because neither the maximum turning rate nor directionality was selected by the model selection procedure.

All the analyses were carried out using R 3.3.2 (The R Foundation for Statistical Computing, Vienna, Austria) with the package *gamm4* for GAMM, and the package *lme4* for the mixed effects logistic regression analysis.

## Supplementary Material

Supplementary information
