# Correction: Effect of initial body orientation on escape probability of prey fish escaping from predators (doi:10.1242/bio.023812)

**DOI:** 10.1242/bio.045997

**Published:** 2019-07-15

**Authors:** Hibiki Kimura, Yuuki Kawabata

There were errors in Biology Open (2018) 7, bio023812 (doi:10.1242/bio.023812).

The authors contacted the journal to make us aware of issues with the data presented. Specifically, they mistakenly included some data that should have been excluded (based on distance to the wall and prey swimming behavior) and excluded other data that should have been included. Additionally, some other data were miscategorized (‘capture’ versus ‘escape’).

The authors reanalyzed all the data, which showed that the overall results and conclusions were unaltered. This was confirmed by a reviewer of the original paper, who was asked to review the corrected version and specifically state whether or not, in their opinion, the conclusions were unchanged.

The editorial policies of BiO state that: “Should an error appear in a published article that affects scientific meaning or author credibility but does not affect the overall results and conclusions of the paper, our policy is to publish a Correction…” and that a Retraction should be published when “…a published paper contain[s] one or more significant errors or inaccuracies that change the overall results and conclusions of the paper…”. We follow the guidelines of the Committee on Publication Ethics (COPE), which state: “Retractions should usually be reserved for publications that are so seriously flawed (for whatever reason) that their findings or conclusions should not be relied upon”.

Given this guidance, the journal has decided to publish this Correction, which we have made as detailed as possible.

Both the online full-text and PDF versions of the article and supplementary information have been updated. The original uncorrected PDFs with the errors highlighted are available as supplementary material to this Correction, so that readers can clearly see where changes have been made.

The authors wish to particularly draw readers' attention to the following changes in trend:

- In both versions, escape probability was the highest at the 120-150° initial orientation, and the peak in escape probability occurred at the intermediate value in the logistic regression curve. This trend was not statistically significant in the original version, but was significant in the corrected version (Fig. 4C).
- In both versions, flight initiation distance [flight initiation distance calculated using the closest margin of the prey's body to the predator's snout (FID_body_), flight initiation distance calculated using the nearer prey's eye (FID_eye_) and flight initiation distance calculated using the prey's center of mass (FID_CM_)] in the largest and smallest initial orientations were smaller than those in the other initial orientations. This trend was detected in the regression curve in the original version, but was not detected in the regression curve in the corrected version (Fig. 5A, Fig. S1A,B). Additionally, there was a significant positive linear relationship between initial orientation and FID_eye_ in the corrected version, but the relationship was not significant in the original version (Fig. S1A).
- In both versions, the apparent looming threshold (ALT) was the largest when the initial orientation was away from predators (150-180°) and the values were similar among the other initial orientations (0-150°). This trend was detected in the regression curve, and was statistically significant in the original version. However, this trend was not detected in the regression curve, and was not statistically significant in the corrected version (Fig. 5B).
- Predator speed was the smallest at the 0-30° initial orientation, and the second smallest at the 90-120° initial orientation in the original version. The speed was the smallest at the 120-150°, and the second smallest at the 0-30° initial orientation on the corrected version (Fig. 5C).
- In both versions, an increase of initial orientation tended to decrease the mean turning rate. This trend was statistically significant in the original version, but was not statistically significant in the corrected version (Fig. 6C; Table 1).

The authors apologise to the journal and readers for these errors.

