# Supplementary material for: Effect of initial body orientation on escape probability of prey fish escaping from predators
Source: Biol Open. 2018 Jun 26;7(7):bio023812. doi: 10.1242/bio.023812 (PMC6078344; doi:10.1242/bio.023812)
Supplement: Supplementary information [file biolopen-7-023812-s1.pdf]

Table S1. Pearson's correlation coefficients for all pairs of continuous explanatory variables.

	FID_{eye}	FID_{CM}	Initial orientation	Maximum turning rate	Predator speed	Relative size
FID_{body}	0.95**	0.95**	-0.11	0.21	0.29	-0.28
FID_{eye}		0.98**	0.00	0.17	0.25	-0.25
FID_{CM}			-0.16	0.22	0.22	-0.22
Initial orientation				-0.32	0.20	-0.20
Maximum turning rate					0.15	0.18
Predator speed						-0.27

FID_{body}, flight initiation distance calculated using the closest margin of the prey's body to the predator's snout; FID_{eye}, flight initiation distance calculated using the nearer prey's eye; FID_{CM}, flight initiation distance calculated using the prey's center of mass
**, $p<0.01$

Biology Open • Supplementary information

Biology Open (2018): doi:10.1242/bio.023812: Supplementary information

Table S2.

No.	FID_{body} (mm)	FID_{eye} (mm)	FID_{CM} (mm)	Initial orientation (°)	Turn angle (°)	Turn duration (s)	Mean turning rate (° s^{-1})	Maximum turning rate (° s^{-1})	Escape trajectory (°)	Directionality	Type of escape response	Predator speed (m s^{-1})	ALT (° s^{-1})	Cumulative distance (mm)	Maximum speed (m s^{-1})	Maximum acceleration (m s^{-2})	Relative size	The prey's body orientation at the start of experiment (°)	Target	Predator ID	Predator depth (mm)	Predator width (mm)	The length between tip and maximum depth on the predator (mm)	Evasion outcome
1	29.03	36.22	46.32	36.86	54.65	0.018	3036.11	4345.87	93.51	away	double bend	0.36	1.52	14.94	0.80	47.63	0.31	173.83	NA	A	41.21	23.87	50.18	escaped
2	50.63	53.19	61.11	36.53	60.38	0.016	3773.98	5045.34	NA	away	double bend	0.65	1.93	10.16	0.63	72.10	0.30	154.80	NA	A	41.21	23.87	50.18	captured
3	9.86	17.75	27.39	22.49	NA	NA	NA	2676.57	NA	toward	NA	0.34	2.26	NA	NA	NA	0.31	180.00	NA	A	41.21	23.87	50.18	captured
4	38.21	37.08	41.51	81.26	47.33	0.018	2629.18	3737.94	128.59	toward	single bend	2.31	9.26	7.57	0.41	49.01	0.27	13.40	0.68	B	38.91	24.56	50.42	escaped
5	75.47	93.51	92.64	122.54	14.92	0.012	1243.28	1874.22	119.63	toward	double bend	1.75	2.65	18.20	0.99	46.43	0.27	172.20	NA	B	38.91	24.56	50.42	escaped
6	0.00	0.00	0.00	165.69	NA	NA	NA	NA	NA	NA	NA	2.25	25.54	NA	NA	NA	0.30	145.50	0.38	B	38.91	24.56	50.42	captured
7	0.00	0.00	0.00	33.38	NA	NA	NA	NA	NA	NA	NA	1.10	12.50	NA	NA	NA	0.26	108.34	0.22	B	38.91	24.56	50.42	captured
8	50.63	44.90	58.51	30.43	NA	NA	NA	5008.65	NA	away	NA	1.61	5.46	NA	NA	NA	0.36	7.47	NA	B	38.91	24.56	50.42	captured
9	92.34	98.00	107.59	35.95	62.33	0.02	3116.55	4525.79	86.77	away	double bend	1.53	2.18	19.05	1.08	66.06	0.34	26.38	NA	B	38.91	24.56	50.42	escaped
10	93.56	89.32	101.58	12.70	86.48	0.024	3603.14	4071.71	276.17	toward	double bend	1.69	2.71	14.41	1.05	85.35	0.49	78.19	NA	B	38.91	24.56	50.42	escaped
11	12.02	4.96	19.02	24.69	NA	NA	NA	NA	NA	away	NA	0.67	6.44	NA	NA	NA	0.43	77.11	0.06	B	38.91	24.56	50.42	captured
12	0.00	0.00	0.00	19.92	NA	NA	NA	NA	NA	NA	NA	0.69	7.88	NA	NA	NA	0.44	180.00	0.44	B	38.91	24.56	50.42	captured
13	8.51	38.93	30.21	149.02	NA	NA	NA	NA	NA	toward	NA	1.96	7.55	NA	NA	NA	0.39	22.69	0.29	B	38.91	24.56	50.42	captured
14	59.67	60.94	71.34	44.50	81.41	0.02	4070.73	5409.83	NA	away	double bend	1.95	4.90	14.71	0.80	72.24	0.32	122.68	NA	B	38.91	24.56	50.42	escaped
15	38.07	39.76	45.39	69.35	NA	NA	NA	5996.04	NA	away	NA	1.46	5.52	NA	NA	NA	0.38	123.29	0.12	B	38.91	24.56	50.42	captured
16	94.91	94.52	101.65	64.60	79.25	0.022	3602.12	5158.85	150.1	away	double bend	1.43	2.14	12.57	0.68	60.80	0.31	35.44	NA	B	38.91	24.56	50.42	escaped
17	38.61	48.24	50.29	101.09	NA	NA	NA	2085.05	NA	toward	NA	1.69	5.38	NA	NA	NA	0.30	161.20	0.27	B	38.91	24.56	50.42	captured
18	108.81	110.20	114.93	90.45	42.4	0.018	2355.81	3307.21	132.85	away	single bend	1.75	2.13	5.84	0.31	17.52	0.29	25.43	NA	B	38.91	24.56	50.42	escaped
19	72.63	70.95	78.80	73.94	55.69	0.018	3093.66	5090.77	NA	away	NA	1.47	3.11	11.21	0.60	46.53	0.26	5.78	NA	B	38.91	24.56	50.42	captured
20	19.04	39.87	36.26	152.22	NA	NA	NA	NA	NA	toward	double bend	0.64	2.50	NA	NA	NA	0.34	69.98	-0.26	C	41.25	24.87	50.42	captured
21	65.21	68.82	76.16	17.18	NA	NA	NA	6194.2	NA	away	NA	1.82	4.15	NA	NA	NA	0.30	106.59	NA	C	41.25	24.87	50.42	captured
22	32.67	31.35	39.16	64.15	NA	NA	NA	5408.3	NA	away	NA	1.45	6.91	NA	NA	NA	0.45	94.08	0.15	C	41.25	24.87	50.42	captured
23	39.29	41.22	49.07	63.51	NA	NA	NA	4580.08	NA	away	NA	1.16	4.44	NA	NA	NA	0.41	14.22	NA	C	41.25	24.87	50.42	captured
24	91.94	93.67	100.59	83.39	134.71	0.028	4811.01	7100.45	218.1	away	single bend	1.84	2.89	17.55	0.73	48.80	0.45	166.59	NA	C	41.25	24.87	50.42	escaped
25	88.56	85.99	89.30	96.58	55.59	0.016	3474.47	4402.56	162.54	away	double bend	1.61	2.82	19.96	1.02	78.10	0.32	52.63	NA	C	41.25	24.87	50.42	escaped

26	32.40	38.99	46.79	75.16	NA	NA	NA	2686.14	NA	toward	NA	1.56	6.23	NA	NA	NA	0.40	103.82	NA	C	41.25	24.87	50.42	captured
27	30.78	23.93	27.17	89.33	NA	NA	NA	NA	NA	away	NA	1.44	8.03	NA	NA	NA	0.37	25.13	0.03	D	43.68	26.31	53.30	captured
28	29.03	26.74	27.17	110.43	NA	NA	NA	NA	NA	away	NA	0.39	2.05	NA	NA	NA	0.40	108.43	0.20	D	43.68	26.31	53.30	captured
29	47.52	63.94	61.69	126.87	33.77	0.016	2110.46	4291.89	86.4	toward	double bend	0.83	1.99	20.22	0.87	35.82	0.31	16.58	NA	E	41.22	24.04	51.33	escaped
30	107.33	114.31	122.53	30.02	106.44	0.024	4434.98	5911.82	136.46	away	single bend	1.23	1.44	16.92	1.09	118.10	0.28	90.26	NA	E	41.22	24.04	51.33	escaped
31	15.39	23.88	37.61	23.72	NA	NA	NA	5610.22	NA	away	NA	1.92	10.57	NA	NA	NA	0.36	119.16	0.11	E	41.22	24.04	51.33	captured
32	21.20	17.26	26.99	3.20	NA	NA	NA	4482.85	NA	toward	NA	2.08	13.66	NA	NA	NA	0.40	134.19	0.42	E	41.22	24.04	51.33	captured
33	52.65	44.84	50.55	84.08	41.78	0.024	1740.87	2703.94	125.86	away	single bend	1.63	5.57	7.43	0.47	55.16	0.32	105.52	NA	E	41.22	24.04	51.33	escaped
34	24.03	40.96	33.52	165.39	38.48	0.012	3206.3	3668.51	205.34	away	double bend	2.16	8.02	28.69	1.17	67.11	0.36	NA	NA	E	41.22	24.04	51.33	escaped
35	60.35	66.42	74.21	6.61	105.88	0.022	4812.7	6366.12	112.49	away	single bend	1.42	3.28	19.12	1.13	66.55	0.43	108.54	NA	E	41.22	24.04	51.33	escaped
36	53.33	61.38	61.38	104.97	76.11	0.018	4228.57	8419.23	181.08	away	single bend	1.49	3.74	30.25	2.43	304.87	0.42	176.49	NA	E	41.22	24.04	51.33	escaped
37	56.97	65.25	77.59	5.37	98.96	0.026	3805.96	5166.42	NA	toward	double bend	1.00	2.36	14.93	0.77	60.40	0.40	171.61	NA	E	41.22	24.04	51.33	captured
38	51.30	58.01	58.22	114.95	85.86	0.02	4293.17	6030.33	212.13	away	double bend	0.92	2.44	15.57	0.87	63.78	0.32	40.43	NA	E	41.22	24.04	51.33	escaped
39	15.12	11.34	22.17	37.02	NA	NA	NA	4833.95	NA	away	NA	1.77	17.00	NA	NA	NA	0.38	167.58	-0.21	F	30.76	19.53	38.26	captured
40	10.26	46.47	69.21	11.24	NA	NA	NA	NA	NA	away	NA	0.44	1.50	0.00	0.00	0.00	0.48	26.03	NA	F	30.76	19.53	38.26	captured
41	10.53	29.91	22.85	156.70	NA	NA	NA	NA	NA	away	NA	1.70	8.88	26.80	1.16	100.54	0.40	84.56	0.50	F	30.76	19.53	38.26	captured
42	18.90	23.03	23.09	106.45	39.36	0.038	1035.75	1345.17	62.16	away	double bend	0.23	1.51	6.35	0.47	28.50	0.46	86.95	NA	F	30.76	19.53	38.26	escaped
43	4.86	6.74	12.84	22.37	NA	NA	NA	NA	NA	toward	NA	0.28	3.17	NA	NA	NA	0.52	56.12	0.08	F	30.76	19.53	38.26	captured
44	70.61	69.38	77.01	49.91	67.76	0.018	3764.51	4693.45	117.67	away	single bend	1.61	3.42	8.71	0.48	26.89	0.36	49.93	NA	G	32.74	19.47	40.66	escaped
45	19.44	34.27	29.11	136.24	27.57	0.01	2756.92	3722.42	142.18	toward	double bend	0.92	4.14	17.29	0.89	147.14	0.39	128.92	2.49	G	32.74	19.47	40.66	escaped
46	69.66	80.49	80.57	112.15	20.71	0.01	2071.32	2627.04	106.6	away	double bend	1.55	2.72	14.80	0.88	49.81	0.36	NA	NA	G	32.74	19.47	40.66	captured

Biology Open • Supplementary information

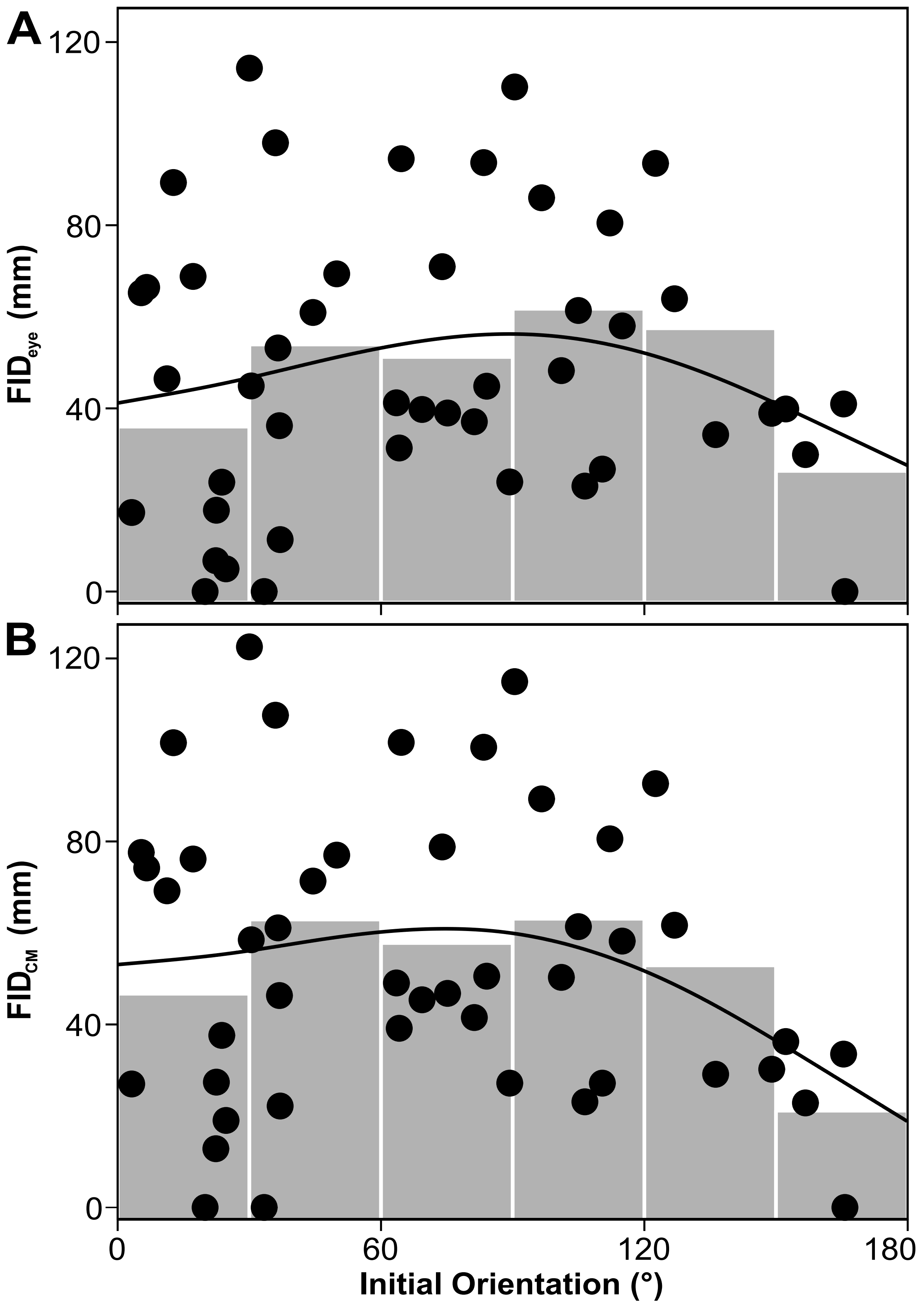
A
B
120
80
40
0
FIDeye (mm)
FIDCM (mm)
0
60
120
180
Initial Orientation (°)

Fig. S1. (A) Relationship between initial orientation and flight initiation distance calculated using the nearer prey's eye (FID_{eye}) (GAMM; F=0.07, P=0.84). (B) Relationship between initial orientation and flight initiation distance calculated using the prey's center of mass (FID_{CM}) (GAMM; F=1.66, P=0.30). These lines were estimated by the generalized additive mixed model. All the prey fish were used in these analyses (n=46). The grey bars represent the mean values for the 30° initial orientation bins.

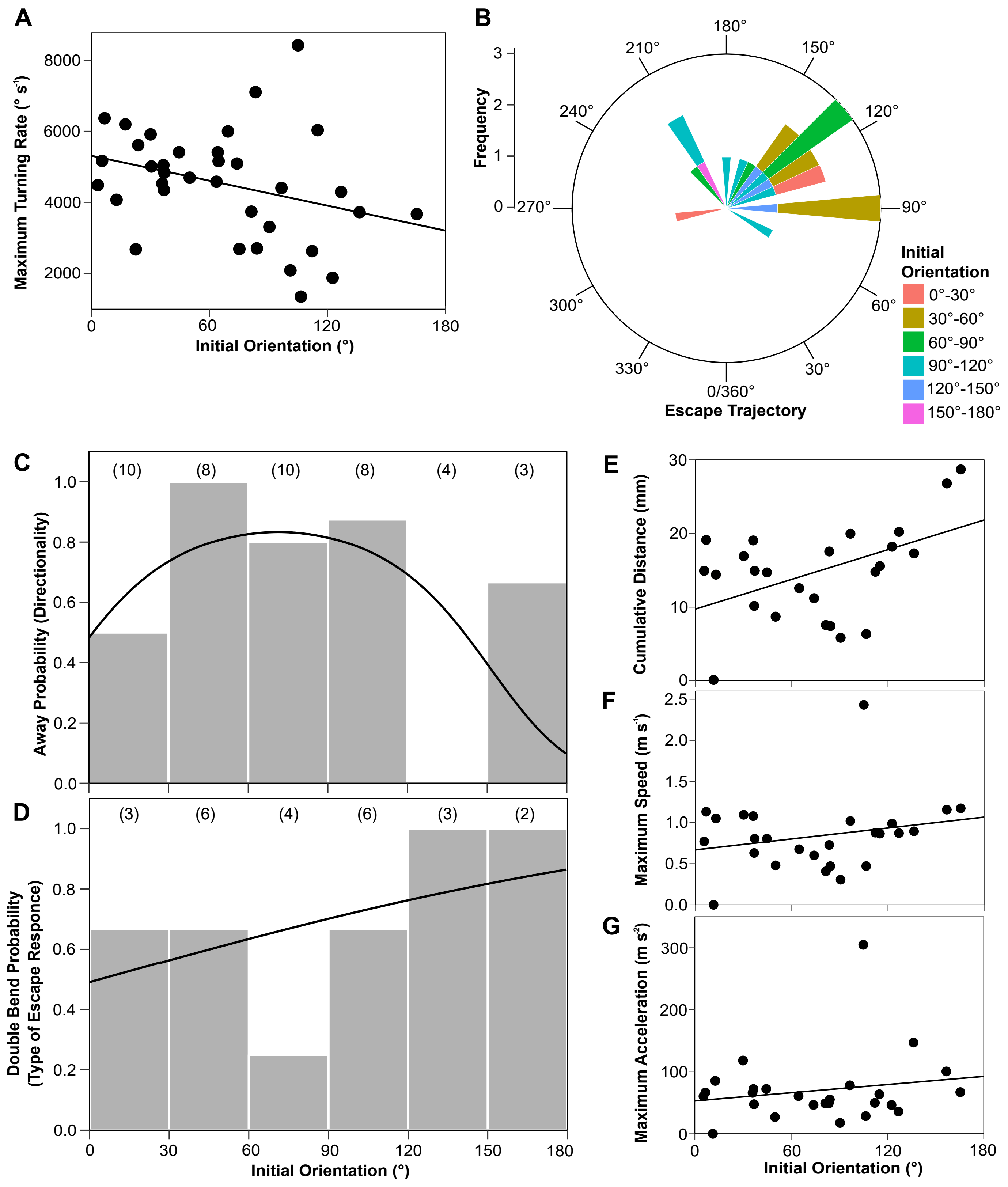

A
Maximum Turning Rate (° s-1)
Initial Orientation (°)
B
Frequency
180°
150°
120°
90°
60°
30°
0/360°
330°
300°
270°
240°
210°
Escape Trajectory
Initial Orientation
0°-30°
30°-60°
60°-90°
90°-120°
120°-150°
150°-180°
C
Away Probability (Directionality)
(10) (8) (10) (8) (4) (3)
D
Double Bend Probability (Type of Escape Responce)
(3) (6) (4) (6) (3) (2)
Initial Orientation (°)
E
Cumulative Distance (mm)
F
Maximum Speed (m s-1)
G
Maximum Acceleration (m s-2)
Initial Orientation (°)

Fig. S2. (A) Relationship between initial orientation and maximum turning rate (n=35, R=-0.32, P=0.06). (B) Relationship between initial orientation and escape trajectory (n=20, Rs=1.94, P=0.38). (C) Relationship between initial orientation and directionality (n=43, χ^2=0.42, P=0.51). (D) Relationship between initial orientation and type of escape response (n=24 χ^2=0.97, P=0.32). (E) Relationship between initial orientation and cumulative distance (n=26, R=0.45, P<0.05). (F) Relationship between initial orientation and maximum speed (n=26, R=0.24, P=0.24). (G) Relationship between initial orientation and maximum acceleration (n=26, R=0.18, P=0.38).

Biology Open • Supplementary information
